# Response of plant nutrient stoichiometry to fertilization varied with plant tissues in a tropical forest

**DOI:** 10.1038/srep14605

**Published:** 2015-09-29

**Authors:** Qifeng Mo, Bi Zou, Yingwen Li, Yao Chen, Weixin Zhang, Rong Mao, Yongzhen Ding, Jun Wang, Xiankai Lu, Xiaobo Li, Jianwu Tang, Zhian Li, Faming Wang

**Affiliations:** 1 Key Laboratory of Vegetation Restoration and Management of Degraded Ecosystems, South China Botanical Garden, Chinese Academy of Sciences, Guangzhou 510650, China; 2 University of Chinese Academy of Sciences, Beijing 100049, China; 3 Xiaoliang Research Station for Tropical Coastal Ecosystems, Maoming 525029, China; 4 Northeast Institute of Geography and Agroecology, Chinese Academy of Sciences, Changchun 130102, China; 5 Agro-Environmental Protection Institute, 300191 Tianjin, P.R. China; 6 Ecosystems Center, Marine Biological Laboratory, Woods Hole, MA, 02543, USA

## Abstract

Plant N:P ratios are widely used as indices of nutrient limitation in terrestrial ecosystems, but the response of these metrics in different plant tissues to altered N and P availability and their interactions remains largely unclear. We evaluated changes in N and P concentrations, N:P ratios of new leaves (<1 yr), older leaves (>1 yr), stems and mixed fine roots of seven species after 3-years of an N and P addition experiment in a tropical forest. Nitrogen addition only increased fine root N concentrations. P addition increased P concentrations among all tissues. The N × P interaction reduced leaf and stem P concentrations, suggesting a negative effect of N addition on P concentrations under P addition. The reliability of using nutrient ratios as indices of soil nutrient availability varied with tissues: the stoichiometric metrics of stems and older leaves were more responsive indicators of changed soil nutrient availability than those of new leaves and fine roots. However, leaf N:P ratios can be a useful indicator of inter-specific variation in plant response to nutrients availability. This study suggests that older leaf is a better choice than other tissues in the assessment of soil nutrient status and predicting plant response to altered nutrients using nutrients ratios.

Nitrogen (N) and phosphorus (P) are commonly found to be the limiting elements for the primary production since they are essential nutrients for plant growth^1^,^2^. The response of plants to atmospheric deposition, climate change, and other human influences will therefore be mediated by changes in the availability of these elements^3^. Previous studies indicated that biological processes in many ecosystems with young soils might be limited by the low supply of N, while those with old soils might be limited by P^2^. The P limitation occurred because P was primarily derived from rock weathering, and P loss from the soil runoff could hardly be compensated by other pathways^4^. Due to increasing fuel combustion and human N fixation worldwide, more and more ecosystems are facing a substantial increase of N inputs. Therefore, phosphorus limitation could be more widespread than generally acknowledged in a changing world.

Tropical forests occur on highly weathered soils, where much of the original P-rich parent material has been lost, and most of the remaining P is occluded on iron and aluminum oxides^5^. Therefore, it is generally believed that plant growth might be limited by P in tropical forests^6^,^7^,^8^. However, a meta-analysis of nutrient addition experiments suggested that N limitation was equally strong for temperate and tropical forests^9^. Recent studies also suggested that nutrient limitation was complex and often regulated by several interacting resources, collectively regarded as the multiple limitation resources theory^10^,^11^. For example, in a tropical forest, seedling growth rate was limited by multiple nutrients (N, P and K) and their interaction^12^. These conflicting results reflect an uncertainty in the nutrient limitation of tropical forests. Therefore, a better understanding of nutrient limitation in tropical forests may be critical for mitigating climate change, since tropical forests play an important role in the global carbon (C) cycle^13^.

Examining plant tissues nutrient concentrations and N:P ratios within the same species in experimental N and/or P addition sites is a useful approach to evaluate the nutrient status of ecosystems^14^,^15^, since the variations of N:P ratios generally could be interpreted by the supply of nutrients. However, only a handful of field fertilization studies have been conducted in tropical forests, and most of these studies only reported plant growth rates. For example, Alvarez-Clare *et al.*^16^ found that trees per plot increased basal area with P addition after 2.7 years fertilization in a tropical forest of Central America, indicating a P limitation of this tropical forest. Denslow *et al.*^17^ reported that added N also increased the tree trunk growth in a Hawaiian young tropical forest. More recently, Ceccon *et al.*^18^ addressed seedling growth in response to nutrient addition in two Mexican tropical forests and observed increased seedling growth with N addition in a young secondary forest and with P addition in a 60-year-old forest. Santiago *et al.*^12^ suggested that the seedling growth rate in Panamanian tropical forests was limited by multiple nutrients. However, seedlings may have different nutrient requirements than their adult counterparts. These results indicated that our knowledge of limiting nutrients in tropical forests was still rare and inconsistent.

Plant nutrient concentrations and their stoichiometric ratios were widely used as indicators for plant nutrient status, and nutrient supply of ecosystems^19^. However, previous studies mainly focused on new green leaves^12^,^20^, while neglecting the responses of other plant tissues, such as older leaves, stems and fine roots to experimental N and P addition^21^,^22^. Actually, due to the stronger homeostatic control over N:P ratios in new leaves than in older leaves or stems^23^, the allocation or storage of excess N or P uptake from soils could preferably occur in stems or older leaves rather than in new leaves when only one of these nutrients were limiting^24^,^25^. Moreover, the active new leaves could also acquire nutrients through the nutrient resorption or movement within plants^24^. The N:P ratios in new leaves thus would be less responsive to altered soil nutrients availability than those in other plant tissues^22^. Therefore, it is necessary to investigate the responses of different plant tissues to altered soil N or P availability due to environmental changes.

Most tropical forest fertilization experiments were conducted in tropical America or the Hawaiian islands, though similar studies rarely occurred in Asia^26^, where about 393 million ha of tropical forests exist^27^. Tropical Asia, one of the five major regions of tropical forests worldwide (including tropical America, Africa, Asia, Madagascar island and New Guinea), has its distinct ecological traits and species composition^28^. For example, the understory of Asian tropical forests is generally dominated by sterile saplings of canopy trees, whereas that of the neotropics is rich in fruiting shrubs and small trees^29^. This difference not only affects the understory frugivores, but also had a critical role in canopy trees regeneration. These differences also suggest that plant community in Asian tropical forest may have different response to nutrient fertilization as those in the neotropics. Therefore, equally assessing the response of Asian tropical forests to fertilization is valuable for our understanding of tropical forests.

Southern China, located in subtropical and tropical Asia, has a large area of tropical forests, most of which are secondary forests, which were established on previously deforested land^30^. Due to fast economic development, this region is suffering from serious atmosphere N deposition^31^. Here, we report N and P concentrations and N:P ratios in new leaves, older leaves and stems of seven species and mixed fine roots after three years of N and P fertilization in a secondary tropical forest. We expect that: 1) due to high N deposition in this region, the forest should be P limited; and thus N addition would have a relatively weak effect on N concentrations and N:P ratios while P addition would dramatically increase/decrease P concentrations/N:P ratios of plant tissues; 2) N and P concentrations would vary among different plant tissues, and the values in older leaves, stems and fine roots would be more greatly altered by changed nutrient status than those in new leaves.

## Results

### Nitrogen (N) and Phosphorus (P) concentrations in new leaves, older leaves and stems

After several years N and P fertilization, the soil available N and P were significantly increased by N and P addition (Table 1). In the seven species, plant N concentrations showed a consistent pattern in different tissues, with much higher value in new and older leaves than that in stems (Fig. S1). Moreover, N concentrations in new leaves, older leaves and stems exhibited consistent species variation (Table 2, *P* < 0.001 for all), with the values ranging from 13 mg g^−1^ to 18 mg g^−1^, and *Carallia brachiata* showed higher N concentrations than other species (Fig. S1). Nitrogen concentrations of new leaves, older leaves and stems were neither significantly altered by N addition nor P addition (Table 2, *P* > 0.05 for three tissues). In +N plots, N concentrations in new leaves and older leaves were slightly higher (5%–20%) than those in control treatment (CT) in four species (Fig. S2 a & b). There was a 10–80% increase of stem N concentrations in +N plots compared with CT in four out of seven species (Fig. S1 a, c, d & g). However, in the remaining three species (*Syzygium hance, Syzygium bullockii and Canthhium horridum*), stem N concentrations had negative response to N addition (Fig. S1 b, e & f).

**Figure 1:**
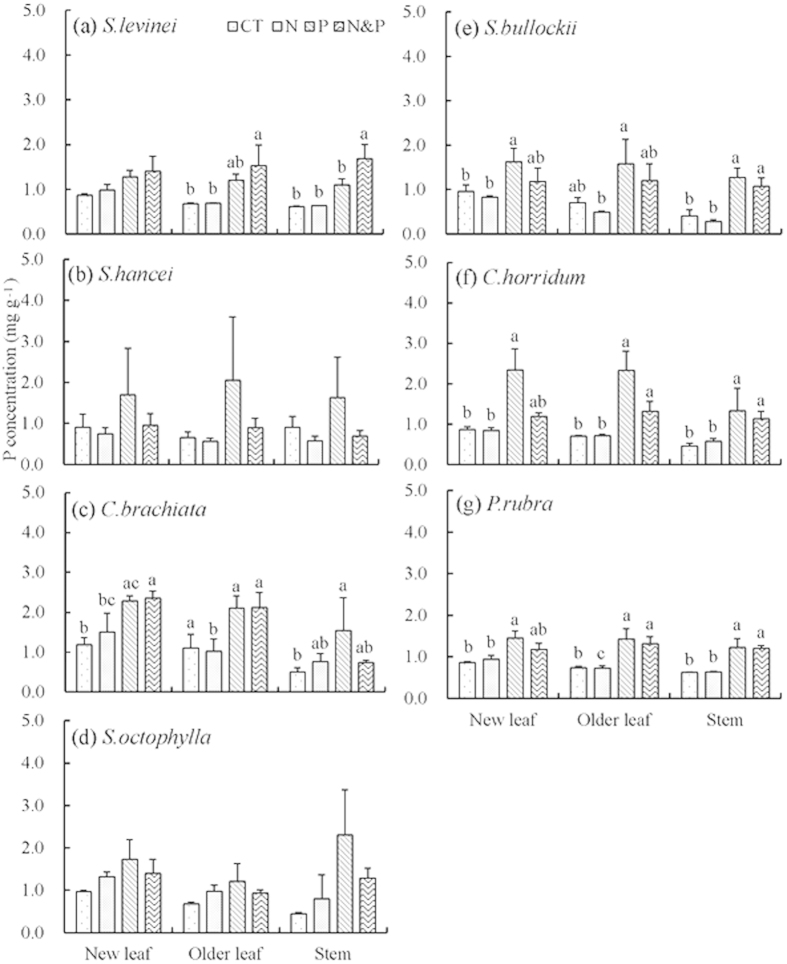
Phosphorus (P) concentrations (mg g^−1^) in plant tissues of seven tree species exposed to N and P addition. Error bars represent standard error. Different lowercase letters denote significant differences between treatments for plant tissue of each species.

**Figure 2:**
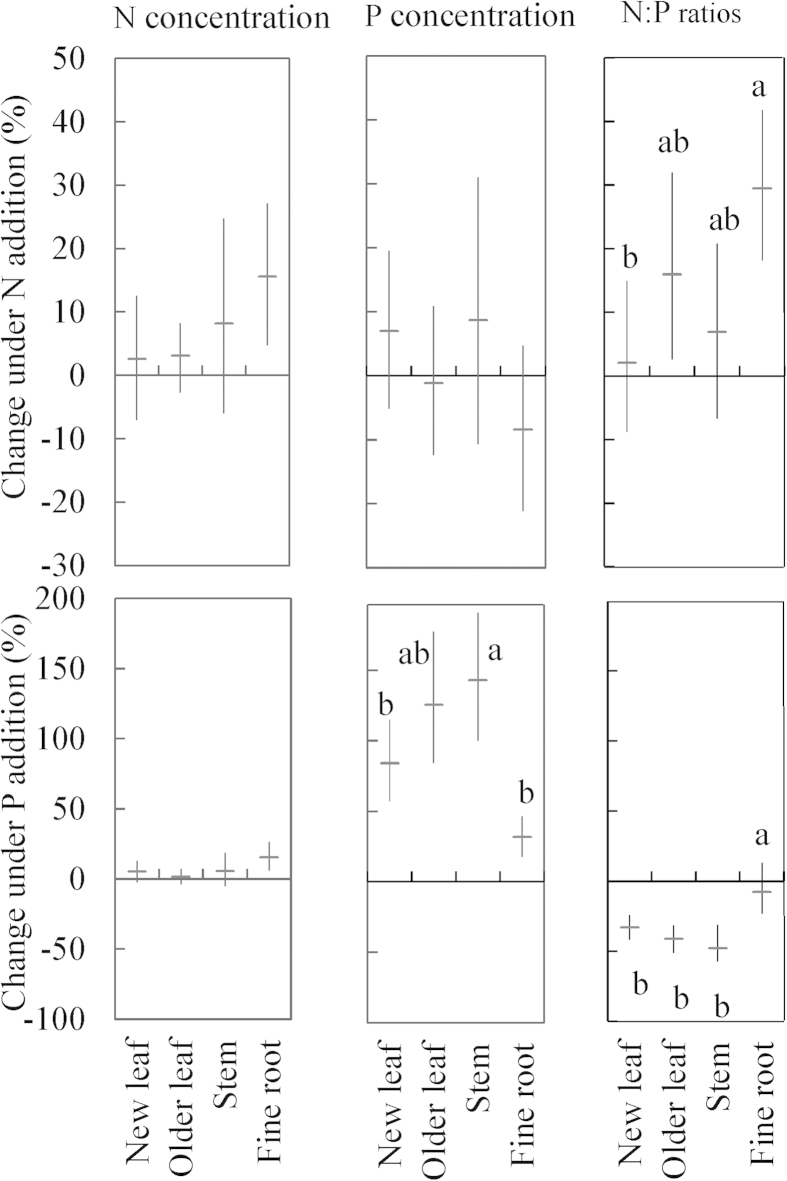
The relative effect (RE) of N concentration, P concentration and N:P ratios in response to N and P addition in different plant tissues. Error bars indicate statistical 95% confidence interval in the figure. Different lowercase letters denote significant differences response to N addition or P addition between four plant tissues.

Plant tissue P concentrations had the same pattern as N concentrations among different tissues (Fig. 1). Nitrogen addition did not significantly affect P concentrations in new leaves and older leaves (Table 2), but significantly affected P concentrations in stems (*P* = 0.03, Table 2). Compared with CT plot, +N plots had 2%–80% higher stem P concentrations in five species (Fig. S3 c). Furthermore, the +NP plots had lower plant P concentrations than +P plots in all species except *Syzygium levinei* (Fig. 1 & Fig. S3), suggesting significant interaction of N × P on P concentration (Table 2, *P* = 0.01, 0.07 and 0.02 for new leaves, older leaves and stems, respectively). P addition significantly increased P concentrations in new leaves, older leaves and stems (Table 2, *P* < 0.001 for all three tissues). In +P plots, the P concentrations were significantly increased by 57%–169% in new leaves, 51%–228% in older leaves and 63%–232% in stems for all seven species relative to CT plot (Fig. S3). The relative effect (compared to the CT, RE) of P concentrations in +P plots in stems was generally higher than the corresponding value in new leaves and older leaves (Fig. 2), suggesting a greater variation of P concentrations in stems than that in leaves in response to P addition.

**Figure 3:**
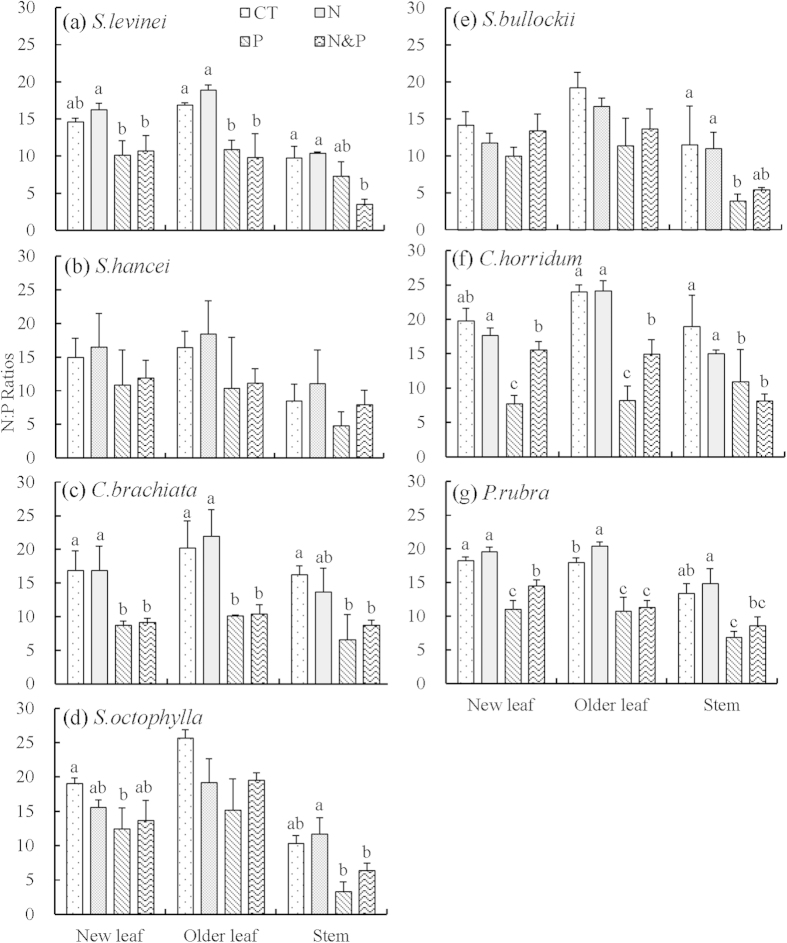
N:P ratios in plant tissues of seven tree species exposed to N and P addition. Error bars represent standard error. Different lowercase letters denote significant differences between treatments for plant tissue of each species.

Species had a significant effect on plant tissue N:P ratios (Table 2, *P* < 0.01), with the values ranged from 15 to 21 in new and older leaves and from 10 to 18 in stems among the seven species (Fig. 3). Nitrogen addition significantly affected the N:P ratios in new leaves and older leaves (Table 2, *P* = 0.01 and *P* = 0.03), but not in stems. However, P addition significantly decreased the N:P ratios in all tissues (Table 2, *P* < 0.001 for all three tissues). Compared to CT, +P plots decreased 16%–61% N:P ratios in new leaves, 21%–66% in older leaves and 18%–65% in stems (Fig. S4). Regardless of plant species, the relative effect of P concentrations after P fertilization was relatively larger in stems and older leaves than that in new leaves (Fig. 2).

### Nitrogen and P concentrations in mixed fine roots

**Figure 4:**
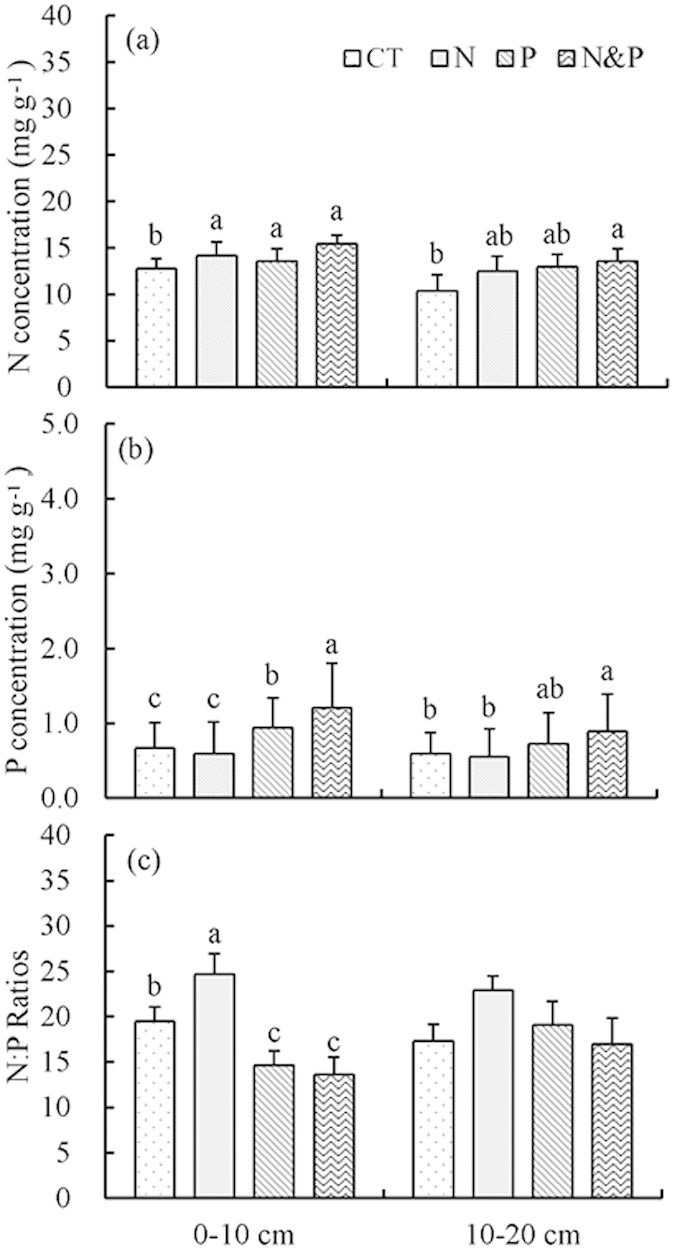
Nitrogen and P concentrations and N:P ratios of mixed fine roots of seven tree species exposed to N and P addition. Error bars represent standard error. Different lowercase letters denote significant differences between treatments for mixed fine roots.

Both N and P addition significantly increased the N concentrations of mixed fine roots (Table 2, *P* = 0.01 for both). In relative to CT, +N plots had 11% higher N concentrations in 0–10 cm layer and 21% higher in 10–20 cm layer, while +P plots had 6% higher N concentrations in 0–10 cm layer and 25% higher in 10–20 cm layer (Fig. 4). P addition greatly increased P concentrations of mixed fine roots relative to CT with the relative effect of P concentrations in +P plots ranging from 41% to 23% in 0–10 cm and 10–20 cm layer, respectively (Table 2, *P* < 0.001, Fig. 2). In mixed fine roots, +N plots had significantly higher N:P ratios than CT, while +P plots did not significantly changed N:P ratios (Fig. 4c).

### The relationship between relative effect of N or P concentrations and plant initial N:P ratios

**Figure 5:**
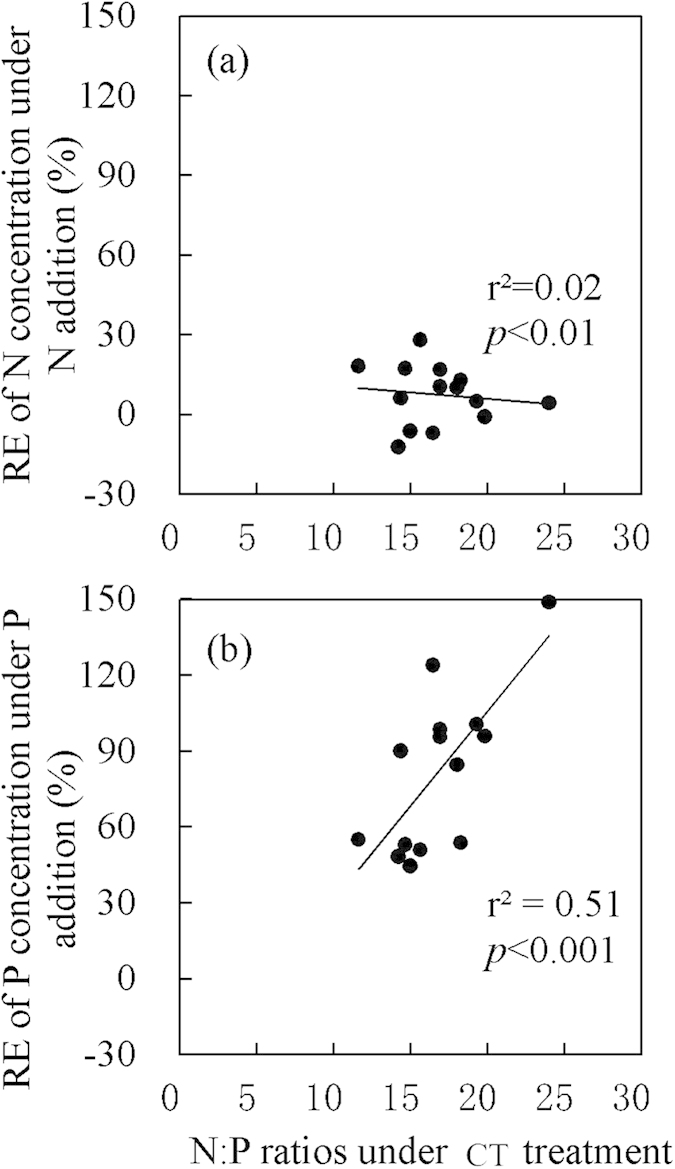
The correlations between plant initial N:P ratios and RE of N or P concentration under N addition and P addition in leaves. The data of new leaves and older leaves were pooled together in this figure because of the similar pattern in both new leaves and older leaves, and the individual data of new leaves or older leaves, stem were also showed in Fig. S5

The connections between initial leaf N:P ratios in the seven species (N:P ratios in CT plots) and its relative effect of N or P concentrations under N or P addition were analyzed (Fig. 5). The relative effect of N concentrations under N addition showed a weak association with plant initial N:P ratios (Fig. 5a, *r*^2^ = 0.02, *P* < 0.01), but the relative effect of P concentrations under P addition was strongly associated with plant leaf N:P ratios (Fig. 5b, *r*^2^ = 0.51, *P* < 0.001). However, we did not observe a similar relationship in stems (Fig. S5).

## Discussion

In this study, new and older leaf N:P ratios among the seven species in CT plots ranged from 15 to 21. If we accepted the concept that N:P ratios below 14 and above 16 indicated N- and P-limited biomass production, respectively^32^,^33^, plant growth would be more controlled by P- than N-limitation in this forest. These results also agreed with our data from vegetation growth in the forest. Specifically, from 2010 to 2013, the highest tree growth rate was with +P plots (52.48 t ha^−1^ year^−1^), followed by +NP plots, +N plots and CT^34^. Furthermore, N concentrations in aboveground tissues (new leaves, older leaves and stems) did not exhibit any significant increases after N fertilization. Conversely, P addition greatly increased P concentrations among all plant species and tissues. This supports the idea that N was not a limiting factor for plant growth because of the increased soil N availability caused by high atmospheric N deposition (40 Kg N ha^−1^ a^−1^ for the wet N deposition) in the studied forest^34^. Collectively, this evidence supports our first hypothesis that this forest is P limited, rather than N limited.

The effect of N addition on leaf and stem N concentrations in the seven species was inconsistent. In +N plots, we only observed a slight increase (5%–20%) in leaf N concentrations than that in CT in five species. This result was consistent with some previous studies in tropical forests, which were usually P-limited^20^,^35^. From an over 15 yrs fertilization experiment in tropical forests, Mayor *et al.*^20^ reported a 9.6% increase of old leaf N concentrations under N addition plots. In this study, the atmosphere deposition data showed that regional wet N deposition was nearly 40 Kg N ha^−1^ yr^−1^ in 2011 and 2012 (unpublished data), which was comparable to the data in many N deposition hotspots worldwide^15^,^31^,^36^. The slight change of leaf N concentrations in +N plots was in line with the idea that tropical trees were usually N sufficient, and did not appreciably accumulate N following fertilization^20^.

Compared to CT, plant P concentrations in +N plots were not significantly altered by N addition across the seven species (Fig. 1). However, N addition greatly reduced P concentrations in +NP plots relative to +P plots in six species, suggesting a negative effect of N addition on plant P concentrations under P addition (N × P interaction: *P* < 0.01, Table 2). Previous studies found that chronic high N deposition might seriously intensify the P deficiency^37^,^38^. In a meta-analysis study, Yuan and Chen^39^ found that N addition decreased leaf P concentrations in non-P-limiting temperate broad-leaf forest. One potential mechanism might be associated with the reduction of arbuscular mycorrorhizal fungi (AMF) abundance after N and P co-addition. In forest soils, AMF performed an important role in plant nutrient and water uptake. Camenzind *et al.*^40^ observed a significant negative effect of N addition on AMF abundance, and the combined treatment with P addition provoked an even stronger decrease (−39%) in AMF richness in a tropical montane forest. In this experiment, although we did not investigate the alteration of AMF after nutrient addition, there was a decrease of soil fungal biomass after N addition^41^. It thus was highly possible that the N and P co-addition leaded to a reduction of soil AMF abundance and richness, which further limited the P uptake to aboveground plant tissues in spite of high soil available P. This assumption was in accordance with the pattern of increased plant growth rates among different treatments (+P plots > +NP plots > +N plots > CT). Although the potential mechanism behind this result still needs further studies to clarify, the implications are profound for deeper studies. Given the dramatic increase of anthropogenic N deposition in tropical forests, the positive effects of P addition on plant growth may be hampered due to the reduction of P uptake.

In herbaceous plants, leaf N:P ratios, especially in recently produced, fully expanded new leaf, is used as target indicators of nutrient availability and limitation to plants^22^. However, when this index is applied to tree species, the nutrient allocation within plant tissues should be carefully considered. New leaf acquires limiting nutrients from other tissues for the maximization of growth, which has been proved by the relocation of N from shaded old leaves to new leaves in several studies^42^. Here, we hypothesized that new leaves would therefore be less sensitive to changes in nutrient status than other plant tissues (i.e. older leaves, stems and fine roots). Our results showed that nutrient addition generally led to a larger variation of N, P concentrations and N:P ratios in older leaves and stems in comparison with these values in new leaves. Phosphorus additions increased P concentrations in new leaves by 84%, by 125% in older leaves, and by 143% in stems among the seven species for average. In response to the increase in plant P concentrations, the average N:P ratios in the seven species decreased in all plant tissues after P fertilization with the order: stems (−47%), older leaves (−41%), new leaves (−33%), and fine root (−7%), as suggested by Garrish *et al.*^23^ in a common garden experiment. Liu *et al.*^21^ also observed stronger effects of N addition on N:P ratios in stems than in leaves in a tropical plant species (*O. pinnata*). Our results were consistent with the findings in Panama tropical forests^22^, and further provided evidence that the survey of nutrient stoichiometry in new leaves of tree species might not provide powerful insight into plant response to altered nutrient status. The metrics of stems and older leaves were more responsive intra-specific indicators of changed soil nutrient availability than those of new leaves.

Recently, Schreeg *et al.*^22^ discussed the inter-tissues variation of N:P stoichiometry within different species, and suggested that stems were more responsive indicators of soil nutrient availability than new leaves. However, this study neglected the substantial inter-specific differences in stem nutrient concentrations. Scaling stem N:P ratios from tissue-level to species-level need inter-specific comparisons. In our study, the relative effect of leaf P concentrations under P addition was highly positively associated with leaf initial N:P ratios (Fig. 5b, *r*^2^ = 0.51, *P* < 0.001). This finding agrees with the prediction that species with higher N:P ratios are more P limited, and thus would have more pronounced response to P fertilization. However, no such relationship was observed in stems (Fig. S5). The result thus indicated that leaf N:P ratio was a more refined indicator in predicting the inter-specific variation of tree species in response to altered soil nutrients. Therefore, considering both inter-tissues and inter-specific variations, we recommend that older leaves would be a better choice than new leaves or stems in the assessment of soil nutrient status and predicting plant response to altered nutrients using tissue nutrients ratios.

Fine roots, which have similar metabolic activities belowground as green leaves do aboveground, were expected to have a similar N to P stoichiometry response to nutrient availability. However, the N and P stoichiometry in fine roots was inconsistent with our predictions. In the study, the response of fine root N:P ratios to nutrient addition was more homeostatic and different from plant leaves and stems. In response to the increased N concentrations, the fine root N:P ratio was significantly increased in +N plots. The fact that fine roots were more constrained by N when compared to leaves and stems makes sense, as fine roots are highly metabolically active and require substantial allocation of N to the synthesis of carrier enzymes that actively acquire nutrients from soil solution^43^. A previous study reported that plant roots appeared to allocate excess N to construction of extracellular phosphatases to acquire P^43^. This increase in phosphatase production with N fertilization implies that even P-limited systems might respond to N deposition with greater productivity, which had been confirmed by our plant growth data (+N plots also had higher plant growth rate relative to control)^34^. Overall, these results reveal that using fine root N to P stoichiometry as an indicator of soil nutrients status is an area in need of more examination.

## Conclusions

In this secondary tropical forest, P addition increased aboveground plant P concentrations, and simultaneously decreased N:P ratios, while N addition had no effect on N concentrations, indicating more intense limitation by P rather than N. We also observed that N addition greatly reduced the P concentrations in +NP plots relative to +P plots in six species, suggesting a negative effect of N addition on the plant P concentrations under P addition. This decline of plant tissue P concentrations might be due to a decrease of soil AMF abundance and richness after N and P co-addition, as suggested by Camenzind *et al.*^40^ in a tropical montane forest. Moreover, we found that stems and older leaves had a more pronounced response to P addition than new leaves, and the response of leaf P concentration to P addition was highly positively associated with initial leaf N:P ratios. The latter finding agreed with the prediction that species with higher N:P ratios would be more P-limited. Finally, this study suggested that the response of fine roots to nutrient fertilization differed from the leaves and stems, suggesting that the use of fine root N to P stoichiometry as an indicator of soil nutrient status needs to be approached with careful consideration.

Based on our findings, we showed that the response of plant nutrient stoichiometry to N and P fertilization varied with tissues and N and P interactions. Given projected increasing N deposition in tropical forest regions, the positive effects of P addition on plant growth would be diminished due to the reduction of P uptake. In sampling practices, under the consideration of both inter-tissues and inter-specific variation, we recommended that older leaves would be better index than new leaves, stems or fine roots when assessing soil nutrient status and predicting plant response to altered nutrients.

## Methods

### Site description

This study was carried out at Xiaoliang Research Station for Tropical Coastal Ecosystem of Chinese Academy of Sciences (21 °27′N, 110 °54′E), southwest of Guangdong Province, PR. China. This region is characterized by tropical monsoon climate with the mean annual temperature of 23 °C. Annual rainfall ranges from 1400 to 1700 mm with a variation of dry and wet seasons. The wet season is from April to October and the dry season from November to March. The soil is latosol developed from granite^34^. The annually wet N deposition in this region was about 40 kg N ha^−1^ in 2011 and 2012 (Wang *et al.*, unpublished data).

The study was conducted in a restored mixed evergreen broad-leaf forest. The forest started as *Eucalyptus exserta* plantation in 1959, then 312 plant species were introduced between 1964 and 1975^44^. Later, the forest succeeded and naturally colonized species displaced almost all planted species by 1990’s. Now, the most common tree species are: *Castanopsis fissa, Cinnamomum camphora, Carallia brachiata, Aphanamixis polystachya, Ternstroemia pseudoverticillata, Acacia auriculaiformis, Cassia siamea, Albizia procera, Albizia odoratissima, Leucaena leucocephala, Aquilaria sinensis, Chakrasia tabularis, Syzygium levinei, Carallia brachiata, Schefflera heptaphylla, Syzygium hancei, Psychotria rubra* and *Aporusa dioica.* The forest is considered as typical secondary evergreen tropical forest in regard to biodiversity and structure complexity of the forest community^34^.

### Experimental design

An N and P addition experiment was designed as a completely randomized block design and established in the secondary tropical forest in September 2009^45^. Each block was located in a site more than 50 meters apart from each other in the forest. Within each block, four 10 × 10 m plots were established and each plot was surrounded by a 2 m wide buffer strip in each site. Four treatments, N addition (+N), P addition (+P), N addition with P addition (+NP), and control treatment (CT, no addition of mineral nutrients) were assigned randomly to the four plots within each block. Each fertilization treatment had five replicates. Both N and P addition were applied at 100 kg ha^−1^ yr^−1^. Nitrogen was added as ammonium nitrate (NH_4_NO_3_) and phosphorus was added as sodium dihydrogen phosphate (Na_2_HPO_4_). Briefly, in every fertilization event, 476.6 g NH_4_NO_3_ (equal to 166.6 g N) and/or 808 g Na_2_HPO_4_ (equal to 166.6 g P) were dissolved in 30 L ground water and then applied to the corresponding plots near soil surface uniformly using a backpack sprayer. Fertilization was conducted in every two months, 6 times in one year, since September 2009. Thirty liters of ground water was also applied to control plots in each treatment event^34^,^41^. Since the annual precipitation of this region could be up to 1700 mm, the amount of added water in each plots only equals to 0.08% and 0.35% of rainfall inputs in wet and dry seasons in 2011.

### Soil sampling and analysis

Soil was sampled in September 2009 (before fertilization) and June 2012 (33^th^ month after fertilization). From each plot, soil cores (5 cm inner diameter) were taken at 0–10 cm depth from 6 randomly selected locations and combined to one composite sample. The litter layer was removed before the soil core was collected. The sample was sieved by 2-mm mesh size after removing the stones and roots by hand.

Soil moisture content was measured by oven-drying for 24 h at 105 °C. Soil pH was determined in 1:2.5 (w/v) soil solutions. Soil NH_4_^+^-N and NO_3_^−^-N in filtered 2 M KCl-extracts of fresh soil sample were measured with a flow injection auto-analyzer (FIA, Lachat Instruments, USA). Soil available P was extracted with Bray-2 solution and determined by the molybdate blue colorimetric method.

### Plant sample collection and measurements

Seven tree species were selected in this study, including *Syzygium levinei, Syzygium hancei, Psychotria rubra, Carallia brachiata, Canthhium horridum, Syzygium bullockii* and *Schefflera octophylla*, all of them were dungarunga or shrub (less than 6 meters height). Leaves sampling was based on the position, color and texture^12^ then distinguish new leaves and older leaves (>1 yr old). Plant new leaves (<1 yr old), which were fully expanded, locating in the top position of branches or stems^12^, older leaves (the lower position, dark green) and stems were sampled in September 2012. Mixed fine roots (<2 mm) were also sampled directly using a 6 cm diameter stainless steel drill with 3 soil core mixture to determine the N and P concentrations in June 2012. All samples were dried for 72 hours at 70 °C in an oven and then finely ground prior to N and P analyses. Tissue N concentration was determined using the Kjeldahl method^46^. Tissue P concentration was measured photometrically after samples were digested with sulfuric acid (H_2_SO_4_). Mass tissue N:P ratios was used in this study.

### Data analysis

Three-Way ANOVA (species, N addition and P addition) was used to evaluate the single or interactive effects of species and treatment on the N, P concentrations and N:P ratios of new leaves, older leaves, stems. The mixed fine root data was also analyzed by three-way ANOVA with soil depth, N addition and P addition as the main factors. The LSD test was generally used to investigate the difference between each treatments in N, P concentrations or N:P ratios.

To assess the effects of nutrient addition on N or P concentrations and N:P ratios, the relative effect (RE) was quantified by the ratios of the variable in the experimental group to the control group minus one, the statistical data was used in Figs 2 and 5, S2, S3, S4, and S5, one-way ANOVA was used to analyze the relative effect data among different tissues. We used the 95% confidence interval to describe relative effect of N, P concentrations and N:P ratios to nutrients addition among four tree tissues (Fig. 2). The correlations between the initial N:P in CT treatment and the relative of N or P concentrations were also analyzed (Fig. 5). All data were performed using the SPSS 16.0 for windows (SPSS Inc., Chicago, IL). The level of significance used in tests was *P* < 0.05.

## Additional Information

**How to cite this article**: Mo, Q. *et al.* Response of plant nutrient stoichiometry to fertilization varied with plant tissues in a tropical forest. *Sci. Rep.* **5**, 14605; doi: 10.1038/srep14605 (2015).

## Supplementary Material

Supplementary Tables and Figures

## Figures and Tables

**Table 1:** General soil chemical properties (0–10 cm) in September, 2009 and June 2012 (mean ± SE, n = 5).

Sampling Date	Treatment	pH	Soil moisture	NH_4_-N (mg kg^−1^)	NO_3_-N (mg kg^−1^)	Available P (mg kg^−1^)
Sep. 2009	CT	3.99 ± 0.06	0.25 ± 0.01	2.12 ± 0.11	2.88 ± 0.31	4.09 ± 0.56
	N	3.97 ± 0.05	0.25 ± 0.01	1.85 ± 0.10	2.72 ± 0.33	3.79 ± 0.42
	P	3.95 ± 0.05	0.24 ± 0.01	1.81 ± 0.11	2.68 ± 0.34	4.06 ± 0.38
	NP	4.02 ± 0.09	0.25 ± 0.01	2.03 ± 0.17	2.35 ± 0.10	3.70 ± 0.60
Jun. 2012	CT	3.75 ± 0.04	0.26 ± 0.02	3.74^b^ ± 0.94	3.12^b^ ± 0.57	4.22^b^ ± 1.12
	N	3.74 ± 0.05	0.24 ± 0.01	5.44^a^ ± 0.77	4.62^a^ ± 1.11	4.17^b^ ± 1.25
	P	3.82 ± 0.04	0.25 ± 0.02	2.70^b^ ± 0.45	3.36^b^ ± 1.14	38.82^a^ ± 3.42
	NP	3.78 ± 0.05	0.23 ± 0.02	7.01^a^ ± 1.91	6.26^a^ ± 0.78	36.00^a^ ± 4.66

Note: Different lowercase letters denote significant differences between treatments (LSD test, *p* < 0.05).

**Table 2:** The three-way ANOVA analysis (*p*-values) for N, P concentration and N:P ratios in new leaves, older leaves, stems, and mixed fine roots in the secondary tropical forest of South China (S, Species; N, N addition; P, P addition; D, Soil depth).

Plant Tissues	Sources	N concentration	P concentration	N:P Ratios
New leaves	N	0.10	0.07	***0.01***
	P	0.75	***<0.001***	***<0.001***
	S	***<0.001***	***<0.001***	0.06
	N × P	0.06	***0.01***	0.07
	N × S	0.20	0.20	0.66
	S × P	0.75	0.34	0.60
	N × P × S	0.22	0.68	0.21
Older leaves	N	0.15	0.06	***0.03***
	P	0.84	***<0.001***	***<0.001***
	S	***<0.001***	***0.02***	***0.01***
	N × P	0.63	0.07	0.59
	N × S	0.92	0.36	0.61
	S × P	0.94	0.31	0.29
	N × P × S	0.79	0.42	0.14
Stems	N	0.26	***0.03***	0.10
	P	0.35	***<0.001***	***<0.001***
	S	***<0.001***	0.53	***0.01***
	N × P	0.19	***0.02***	0.84
	N × S	0.31	0.12	0.38
	S × P	0.70	0.54	0.13
	N × P × S	0.18	***0.04***	0.44
Mixed fine roots	N	***0.01***	0.16	0.18
	P	***0.01***	***<0.001***	***<0.001***
	D	***0.01***	***0.01***	0.49
	N × P	0.65	***0.02***	***0.02***
	N × D	0.85	0.76	0.91
	D × P	0.50	0.08	***0.04***
	N × P × D	0.38	0.56	0.80
