# Supplementary material for: Response of plant nutrient stoichiometry to fertilization varied with plant tissues in a tropical forest
Source: Sci Rep. 2015 Sep 29;5:14605. doi: 10.1038/srep14605 (PMC4586514; doi:10.1038/srep14605)

**Title:** Response of plant nutrient stoichiometry to fertilization varies with plant tissues in a tropical forest

**Authors:**

Qifeng Mo^1,2,3^, Bi Zou^1,3^, Yingwen Li^1,3^, Yao Chen^1,2^, Weixin Zhang^1,3^, Rong Mao^4^, Yongzhen Ding^5^, Jun Wang^1,3^, Xiankai Lu^1^, Xiaobo Li^1,3^, Jianwu Tang^6^, Zhian Li^1,3*^, Faming Wang^1,3*^

**Affiliations:**

^1^Key Laboratory of Vegetation Restoration and Management of Degraded Ecosystems, South China Botanical Garden, Chinese Academy of Sciences, Guangzhou 510650, China;

^2^University of Chinese Academy of Sciences, Beijing 100049, China

^3^Xiaoliang Research Station for Tropical Coastal Ecosystems, Maoming 525029, China

^4^Northeast Institute of Geography and Agroecology, Chinese Academy of Sciences, Changchun 130102, China

^5^ Agro-Environmental Protection Institute, 300191 Tianjin, P.R. China.

^6^Ecosystems Center, Marine Biological Laboratory, Woods Hole, MA, 02543, USA.

***Corresponding author:**

Dr. Faming Wang, Email: [wangfm@scbg.ac.cn](mailto:wangfm@scbg.ac.cn); phone: +86-20-37252905, Fax: +86-20-37252905 or Dr. Zhian Li.

**Supplemental material**

Fig. S1｜Nitrogen (N) concentrations (mg g^-1^) in plant tissues of seven tree species exposed to N and P addition. Error bars represent standard error. Different lowercase letters denote significant differences between treatments for plant tissue of each species.

Fig. S2｜The species-specific relative effect (RE) of N concentrations in response to nutrient fertilization in new leaf (a), older leaf (b) and stem (c).

Fig. S3｜The species-specific relative effect (RE) of P concentrations in response to nutrient fertilization in new leaf (a), older leaf (b) and stem (c).

Fig. S4｜The species-specific relative effect (RE) of N:P ratios in response to nutrient fertilization in new leaf (a), older leaf (b) and stem (c).

Fig. S5｜The correlations between plant initial N:P ratios and relative effect (RE) of N or P concentrations under N addition and P addition in new leaf (a & b), older leaf (c & d) and stem (e & f).

Fig. S1

Fig. S2

Fig. S3

Fig. S4

Fig. S5


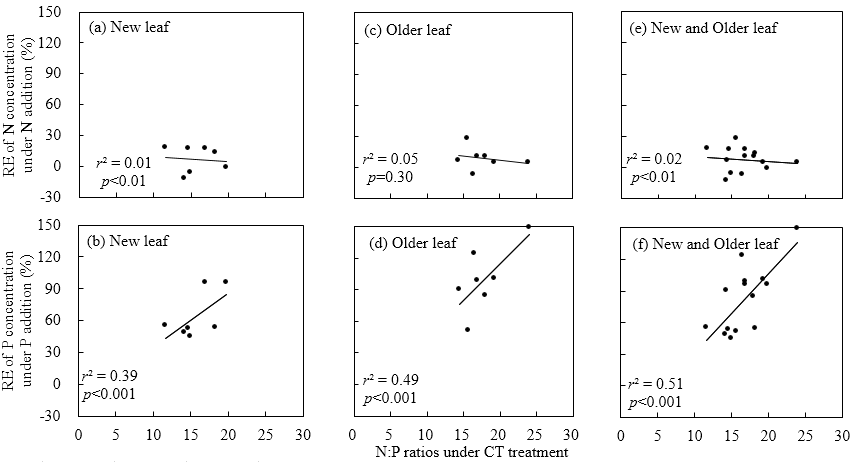

Supplement: Supplementary Tables and Figures [file srep14605-s1.doc]
